# Gender systems in the Putin autocracy

**DOI:** 10.3389/fsoc.2024.1327946

**Published:** 2024-04-04

**Authors:** Elizabeth A. Wood

**Affiliations:** History Faculty, Massachusetts Institute of Technology, Cambridge, MA, United States

**Keywords:** hypermasculinity, homosociality, informal leadership, implicit violence, threat narratives, supporting female roles

## Abstract

Over the last 23 years, Russian President Vladimir Putin’s autocracy has revealed a set of interlocking gender systems that have come to the fore particularly vividly since the full-scale invasion of Ukraine on February 24, 2022. How, this article asks, have the masculinist cultural and political practices of the Putin regime undermined democratic practices and engagement broadly speaking? How have they organized Russian state and society in ways that have led to today’s war in Ukraine with its massive destruction, violence, and brutality? And have there been earlier signals that should have warned observers that this regime might undertake such a war of aggression? Drawing on public, mass media data, this article analyzes the gendered structures of power in Russia that have contributed to the degeneration of democracy in three main areas: (1) male-on-male domination in discourse and practice that supports Putin’s personal rule and emasculates his enemies; (2) the elevation of male power clans, including the President’s personal praetorian guard and the Russian private military companies; and (3) the overall taming and emasculation of the Russian Parliament combined with the elevation of tough women deputies, whom I call the Baba Commissars. These female MPs support the President’s domination by creating an appearance of a threatening outside world that needs to be kept at bay. At the same time, they support a neo-traditional gender order with women managing the house under the direction of the patriarchal male leader.

## Preface: the Prigozhin events of summer 2023 as a microcosm of larger Putin-era gender systems

On June 23–24, 2023 in a major uprising, Yevgeny Prigozhin led his Wagner Private Military Company (PMC) to the Russian city of Rostov-na-Donu, near the Ukrainian front, and his leading commander Dmitry Utkin marched Wagner troops to within 100 km of Moscow. Two months later, Prigozhin and Utkin were presumed dead after the crash of Prigozhin’s private jet. Much has been written about these events, but their structural gender causes and consequences bear additional scrutiny. In a microcosm, this conflict and its ultimate resolution show the importance of examining Russian politics and military on three main levels, all of them highly gendered: (1) macho language; (2) male power clans; and (3) Russian leadership structures predicated on alpha male behaviors governing loyalty and punishment, together with emasculation or elimination of the regime’s enemies.

On social media in the weeks and months before June 23, Prigozhin fulminated in rough, masculine street language reminiscent of Vladimir Putin’s own early macho language use. And he did so for similar reasons—to express his solidarity with his men; to demonstrate his authenticity, and to accentuate his power. At the same time, however, he broke the implicit political rules of who could say what. He referred to the Russian authorities, especially the military authorities who failed to give his Wagner soldiers sufficient ammunition, as “scum,” “paper pushers,” and “bastards” (Velo Daily, 2023). Putin seems to have tolerated this as long as it could serve as a stimulus to his own Ministry of Defense to up their game in fighting Ukraine’s forces. Once it crossed into open rebellion, it became unacceptable.

On the level of clans, Prigozhin had created his own fiefdom in the world of Wagner PMC, the Internet Research Agency (his troll factory), and other outlets. For years Putin maintained conflict and rivalry between multiple clans, including, in this case, between Prigozhin and Sergey Shoigu, the Minister of Defense, who is not widely respected by the military since he has undergone only minimal military training and has not served in actual combat. One of the precipitating causes of Prigozhin’s “March for Justice” to Rostov and Moscow came from Shoigu’s order 2 weeks earlier (on June 10) that all Russian troops fighting in Ukraine would have to sign up for contracts with the Ministry of Defense by July 1. Before the ink had even dried and the order been conveyed, Prigozhin openly rejected it, setting up the conditions for what can only be called (in serious historical nomenclature) “a pissing match” between the two men (Al Jazeera, 2023). Had Prigozhin acceded to Shoigu’s order, he would have undermined the key principles he, as warlord, had used to keep his troops as a significant fighting force: his ability to give his fighters cohesion with personal loyalty to him and his commanders; his ability to pay them so-called “black cash” outside the official military budget of Russia; and his ability to do his own recruiting in prisons and in special recruiting centers throughout Russia (Rainsford, 2023).^1^ Concomitantly, for Shoigu to allow Prigozhin to continue to lob criticisms at the Kremlin and the military from the sidelines was to undermine his own masculine dominance of the military and the *siloviki* (the power structures of Russia which include the secret services as well as the official military).

The inner (masculine) workings of the Russian leadership were on display not only in the conflict between the two coteries (Shoigu’s and Prigozhin’s) and in Putin’s demands for complete loyalty to him personally, but also in the choice of Belarus leader Aleksandr Lukashenko, a lesser male leader, as the principal negotiator with Prigozhin. For weeks before the uprising, Putin had been giving Prigozhin the silent treatment—refusing to take his calls—just as he did in 2015–2016 when he wanted to demean Turkish leader Recep Tayyip Erdogan after the downing of the Russian plane. As the situation unraveled, Putin apparently claimed that he could not be the one to call Prigozhin because he did not think Prigozhin would pick up the phone. In fact, however, he almost certainly delegated the demeaning job of actually entering into negotiations to Lukashenko so he would not himself be compromised or shown to be weak. One Belarusian government propagandist noted the macho posturing of the two men: “They immediately blurted out such vulgar things it would make any mother cry. The conversation was hard, and as I was told, masculine” (Hopkins, 2023).

Two months after the uprising, Prigozhin’s private plane crashed, killing him, Utkin and his security chief. That same day Prigozhin’s ally, General Sergei Surovikin, was dismissed. According to the logic of the system, Prigozhin, Surovikin, and the others had to be sacrificed and even killed to instill fear in the military leadership, the oligarchs, and the private military companies. In Russian historical parlance, this was an “exemplary thrashing” [*pokazatel’naia porka*], a show of extreme punishment to demonstrate to all observers the costs of insubordination. Threats—physical, violent, and often demeaning—are always at the roots of maintaining the male-dominated system.

Stepping back from this incident, one can see that the same hypermasculine language, clan structures, and inner power workings have long been visible in the decades leading up to Russia’s full-scale invasion of Ukraine on February 24, 2022. Examining these phenomena more closely can demonstrate some of the possible signs that war was on the horizon. Of course, hindsight is often 20/20, but in an opaque system like Russia, warning signals are still extremely useful, even after the fact.

## Focus of this article

At the center of this article lie three key questions: How have the masculinist cultural and political practices of the Putin regime undermined democratic practices and engagement broadly speaking? How have they organized Russian state and society in ways that have allowed Putin and his cronies to take the extraordinary decision to invade a sovereign neighbor (Ukraine) with all the attendant massive destruction, violence, and brutality? Have there been earlier signals that should have warned observers that this regime might undertake such a war of aggression?

Scholars have done excellent work on the coercive and personalist nature of this regime (Soldatov and Borogan, 2010; Dawisha, 2011, 2014; Taylor, 2011, 2018; Hale, 2015; Volkov, 2016; Easter, 2017; Rivera and Rivera, 2018; Shamiev and Renz, 2023). Yet the ways in which the Russian autocracy is deeply imbued with masculine behaviors, language, and interconnections has been much less studied. Credit goes to Janet Elise Johnson (2016, 2018) for her pioneering work on the role of women in Russian political institutions, seeming to put forward female candidates but then “boxing them in” so they cannot act independently. Sperling (2015) has shown how Putin’s dominant masculinity is deeply embedded in misogynist thinking inside Russia. My own work has analyzed Putin’s hypermasculinity as a scenario of power, his uses of WWII to create a propaganda bond with his citizenry, and his populist rhetoric as bad boy/good father (Wood, 2011, 2016a; Ekşi and Wood, 2019). Riabov and Riabova (2014) and Ryabova and Ryabov (2011) have explored the many dimensions of the “remasculinization of Russia” in symbolic terms. Scholars have also looked at the conservative turn in Russia and the uses of retrograde social policies relating to gender, especially since 2012 (Sharafutdinova, 2014; Temkina and Zdravomyslova, 2014; Lipman, 2015; Edenborg, 2023; Novitskaya et al., 2023).^2^

In this paper, I focus on how the gendered language, clans, and structures of this regime have created a domestic polity without checks and balances, one prone to excesses and violence. Putin’s hypermasculine political posturing, i.e., his extensive use of political theater in gendered ways, has contributed to the depoliticization and de-democratization of the Russian political sphere. As cultural scholar Kukulin (2018, p. 221) has noted, we can see in this “the state’s annexation of transgression.” As my research here demonstrates, the Russian president’s approach has not only been one of personal hypermasculinity in his immediate circle, but also constant restructuring and restricting of democratic institutions to create a highly masculinized system of control that is enforced by the kingpin alpha male (Putin) personally. At the same time, the Russian Parliament (the Duma), which is officially Russia’s highest legislative authority, has become completely subordinated and “feminized.” Dominant roles are played by a collection of women whom I call the “baba commissars,” i.e., tough women who serve as complements to the Kremlin, augmenting the latter’s messages while emphasizing the external threat to the nation from gays, perverts, and foreigners trying to adopt Russian children. This combination of male inside players and female supporting players strengthens a nondemocratic regime because both groups are deeply invested in supporting a top-down regime. The first (men’s) group does this through male bonding (what sociologists call homosociality; Bird, 1996; Nagel, 2019) and demonstrations of loyalty. The second (women’s) group in Parliament does this through loud demonstrations of support “from below” that sometimes strive to out-Putin Putin. By emphasizing the dangers from nebulous forces that threaten to undermine society, they appeal to the state (both the Kremlin and the Duma itself) to protect and control society for its own good.

Analyzing these gendered patterns of governance—based in male insider politics, violence, and homosociality plus female subordination and amplification of the regime’s messaging—contributes significantly to what sociologist Alena Ledeneva has called “Sistema.” As she (2013, p. 278) shows, Sistema has many aspects, but dominant among them is “the system of governance with its peculiar formal rules and informal norms, combined in a way that is non-transparent for outsiders but recognized by insiders of the public administration in Russia.” Today in Russia, gender in all its multiplicity (discourse, informal clans, and political representation) serves as one of the unspoken pillars of that Sistema with significant consequences for gender relations themselves and for democracy and peace in the region.

## The gendered context in the lead-up to the 2022 full-scale invasion of Ukraine

In the months before Russia’s full-scale invasion, Vladimir Putin and his spokespeople repeatedly showcased his hard, masculine posturing in the Russian political sphere. “Like it or not, you have to put up with it, my beauty,” Putin commented on February 8, 2022 (Putin, 2022). Formally, Putin was criticizing Ukraine for not abiding by the provisions of the Minsk agreement, which, of course, Russia was also not observing. Yet he was also speaking as a man insisting a woman accept his unwanted sexual advances.

For months, Kremlin spokesman Dmitry Peskov had been downplaying Western leaders’ concerns about the Russian build-up in Ukraine by saying that their response was “hysterical,” i.e., emotional, feminized, and overblown (from the Greek word for “womb”) (Radio Free Europe/Radio Liberty 2021; Guy et al., 2022; Slawson et al., 2022). On February 12, Kremlin aide Yury Ushakov told reporters that United States attempts to avert a war in Ukraine were trying to “whip up hysteria” and that “that hysteria has reached its climax” (TASS, 2022a). That same day Foreign Minister Sergey Lavrov also intoned that the West should “get over” its “hysteria” in responding to the impending war (Lemon, 2022; Slawson et al., 2022; TASS, 2022b). Putin himself has often criticized his opponents for overreacting, exhibiting “hysteria” when he does not want them to object to what he himself has imposed. In October 2003, when Russian oligarch Mikhail Khodorkovsky was arrested by 20 gun-toting masked men, Putin called on journalists to “stop the speculation and hysteria” about what had happened (cited in Wood, 2016a, p. 10). The Khodorkovsky case was a leading example of Putin ordering the take-down of a potential rival in a display of militarized masculinity.

Inside Russia the war has been presented in grossly over-the-top masculinized form. YouTube footage has gone viral of a Russian woman who allegedly told her husband to “rape Ukrainian women; just do not tell me about it” (The Telegraph, 2022; Domashchenko, 2023). A Russian Special Operations veteran filmed himself appealing to Putin: “Dear Vladimir Vladimirovich, please decide: Are we fighting a war or jerking off?” (Soldatov, 2022; Soldatov and Borogan, 2022; Youtube, 2022). And of course, sadly, there have been countless examples of brutal, sexualized violence when Russian troops have violated men, women and children (Kulick, 2022; Troianovski, 2022; Horne, 2023). Putin has awarded medals to the Russian military brigade widely believed to have committed some of the worst atrocities in Bucha in April 2022 (Kingsley, 2022; for more on rape in this war, Wamsley, 2022).

When Putin came to power, experts all said that he did not have an ideology. In fact, however, he was very clear that he was creating a “power vertical” [which turned out to mean one-man rule] supported by “sovereign democracy” [i.e., isolationist autocracy]. In classic Putin fashion, this approach hid its true colors for plausible deniability. But his behavior, his policies, and the media reporting on him all converged to create a system based on patriarchy and male violence. Gender scholars sometimes refer to this as “masculinism,” i.e., a dominant gender ideology “capable of setting the terms of normal, just, and proper arrangements for political and social power” and based in male-bonding and male dominance (Duerst-Lahti, 2008, p. 165). One can also think about this as a dominant sociopolitical *practice* (Bourdieu, 2001). It results from powerful men’s proclivities to find like-minded, power-oriented males who share certain informal rules of behavior based on dominance and implied violence. Like a classic protection racket (Tilly, 1985), they must constantly adjust their male behaviors to dominate those below them and curry favor with those above them.

## The hierarchy of gendered structures in the Russian federation

The gendered structures of power in Russia have contributed to the degeneration of democracy on three main levels:

1. the discourse of male domination that supports Putin’s personal rule and emasculates his enemies;
2. the male power clans, especially the informal male leadership of the power ministries, including the President’s personal praetorian guard and the Russian Private Military Companies (PMCs); and
3. the overall taming and emasculation of the Duma (Russian Parliament), combined with the elevation of the Baba Commissars, i.e., those tough women deputies who support the president by creating an appearance of a threatening outside world that needs to be kept at bay.

Analysis of each of these three main areas shows the relevance of the popular Russian proverb “The fish rots from the head.” This holds true not just in the context of corruption (as is usually held), but also in the context of political economy. Putin’s personal machismo, his alpha attacks on his own officials who are demoted to beta status, his creation of a politics of male dominance, his subordination and feminization of all other political institutions, and his defense of Russian imperial dominance have all contributed to a situation where a war against Ukraine, a sovereign, internationally recognized nation of 44 million people, could become thinkable and actionable.

## Putin’s extreme masculinity at the apex

In the Kremlin politics of post-communist Russia, Putin’s person has had an outsized influence as those around him have maneuvered to stand close to power. He is not just occupying an institutional position (the presidency)—he is also recognized as the ground zero of all power. Sycophantic followers have wanted nothing more than to please the leader. They have copied everything from his blue ties (a fashion in the early 2000s) to his manner of speaking. Being close to Putin has meant having access to both power and extraordinary wealth. He stands at the acme of patronal politics, i.e., patron-client relations of mutual feeding and back rubbing (Hale, 2015).

His personal masculinity has influenced the larger political context in two principal ways: (a) his focus on presenting himself as the war president from day 1; (b) his use of derogatory, male slang; and (3) his assumption of a “father” role during the Medvedev presidency (2008–2012) and his continuation of that role in an extremely conservative form in the post-2012 years.

### His posturing as “war president”

From the beginning of his ascent to full power in fall 1999, Putin made winning the second war in Chechnya his signature claim to legitimacy (Wood, 2011, 2016a; Eichler, 2012). He showed that he was willing to carpet bomb Grozny, the capital of Chechnya, in 1999–2002 in order to win, reducing the city and the area around it to rubble; he did the same in Aleppo, Syria in 2016. For Putin, it became more important to win wars than to actually govern and solve social problems. In his *Direct Line* television conversations with his citizens, he adopted the posture of a leader solving the immediate problems of those who petitioned him – an apartment here, a pension there, all without finding systemic solutions that improved the general quality of life (Schuler, 2015). He has consistently done this as a tsar who deigns to answer the people’s pleas through his magnanimity.

The problem with masculinized and militarized posturing is that it assumes that having the leader fly around on military jets—as Putin demonstratively did several times in 1999-2000 before his first presidential election—will bring society together and solve social problems. Political image makers (known as “political technologists” in Russia) felt that solving ordinary problems was not interesting. Rather Russians needed to believe in their leader, in the fight against enemies (Buckley et al., 2023). For 70 years Soviet economics and politics had run on “enthusiasm.” As one political commentator (Rubtsov, 2000) noted in April 2000, “Nothing happens [in Russia] without military adrenaline.” Already at that point, it was clear that Putin was playing on what, in Russian, he called “*beda* [suffering] and *pobeda* [victory].” Lacking “the narcotic of the general struggle,” ordinary people in the post-Soviet period had to come face to face with ordinary life tasks. The real danger, as has since become clear, was that Putin would re-addict them to the “military-political heroin” of jingoism and blind loyalty.

### His use of tough and derogatory language

In order to accentuate his military prowess, Putin relied on tough and derogatory language designed to show that he was a “real man.” His phraseology was one that only men can use and that they principally use in male-only spaces (prisons, the mafia, the military, the bathhouse). “We’ll off them in the outhouse” [*mochit’ v sortire*], he said of the Chechens in fall 1999. But this was not just rude language for its own sake. He was also establishing his dominance over and denigration of others. “It’s unlikely many of you are wearing diapers,” he told an audience of journalists in 2006 after he had been holding court for four hours (Regnum, 2006; Rossiiskaia gazeta, 2006). He effectively threatened a Belgian journalist who asked too many critical questions about Chechnya with circumcision: “Come visit us in Moscow […]. I will recommend they perform an operation so that nothing will grow back” (Kolesnikov, 2002). In this male domination, Putin threatened not only the journalist himself, but also all those who criticized the regime, suggesting that democracy, free speech, and the rule of law were not a priority for the Kremlin at this time.

### The father protector—the nation (Russia) as gendered female, passive and in need of protection

Putin has played a number of different masculine roles. In the first part of his reign, he particularly played the bad boy who would come in and dominate Russian politics, bringing “order and discipline” (his favorite phrases in 1999–2003), but in fact using extra-legal measures and violent language. Then in the years when Dmitry Medvedev was president (2008–2012), Putin played the father/protector who would fly in and resolve any difficult situation. He saved scientists and a television crew from an escaped tiger in Siberia in fall 2008; he descended to the bottom of Baikal in a submersible in 2009; he flew planes over Moscow putting out wildfires in 2010 (Wood, 2008). In the full-scale war against Ukraine, he has managed to play both the bad boy (threatening both Ukraine and the West) and the good father (claiming to protect his nation from threatening outsiders) (Ekşi and Wood, 2019). In the Prigozhin crisis, he fulminated about treason and promised to “protect our people and our state from any threats.” Emphasizing personal dominance has allowed Putin to reinforce an autocratic system based on personal rule at the expense of democratic structures.

## The next layer of the pyramid: the men around Putin

Among Russia experts (Minchenko Consulting, 2012; Johnson, 2016, 2018), it is well known that Putin surrounds himself almost entirely by men. A recent (Snegovaya and Petrov, 2022) study of Russian political elites found that 94% were men; this was true in both 2010 and 2020.^3^ The list of elites includes virtually the entire Presidential Administration, the Security Council, the Prime Minister and members of the government ministries, members of the security forces, the mayor of Moscow and governor of St. Petersburg, the leading figures in Parliament, the leading judicial administration, the super-governors who control Russia’s seven main regions, and the heads of the largest state corporations.

Just below Putin stand the male-dominated, homosocial structures run by and for men and based in male bonding. This includes members of the bureaucracies and the *siloviki*, i.e., the security forces in the military and the intelligence services. If we look at the top power holders who are close to Putin, all are men who have known him for a long time (especially since his time in St. Petersburg, but some, like Arkady Rotenberg, since adolescence), and most are associated with violence, one way or another. Scholars in the field have developed different typologies to talk about their connections and interconnections, grouping them in “clans,” “Kremlin towers,” “Planets,” and an informal “Politburo.” However, as Ledeneva (2013, 32-36, 72-82) persuasively argues in her discussion of *sistema*, their rule has come about through networked allegiances in four main categories: Putin’s inner circle, useful friends, core contacts, and mediated contacts (intermediaries). In virtually all cases it is not their official institutional role, their expertise, or their professionalism that helped them obtain and maintain their positions. Instead, complex systems of unwritten, informal, opaque rules govern the interactions of these top figures.

The gender dimensions of this have not been sufficiently studied, particularly regarding the ways in which *perceived loyalty,* a key requirement for top power holders, is actually based in homosociality, i.e., male bonding rituals of drinking and lounging in saunas, giving out medals in all male-environments, hunting, showing off their mistresses, and the like (Johnson, 2018, 6–7, 32–33, 39–41). At the same time anyone who breaks the rules or steps out of line is confronted with male–male violence and humiliation that can extend to violence against one’s family (Ledeneva, 2013, 240–243). Virtually all of Putin’s inner circle are men who have served as his bodyguards, his coworkers in the secret services, his sparring partners in judo, and his accomplices in the privatization of property and seizure of state wealth. Among the men a variety of groupings (usually referred to as “clans”) have formed and re-formed with continuous infighting at all levels (Kryshtanovskaya and White, 2005; Soldatov and Borogan, 2010; Urban, 2010; Ledeneva, 2013; Dawisha, 2014; Zygar, 2016; Marten, 2017; Reddaway, 2018). Putin’s gender regime both permits men to enact violence against other men and against women and threatens them with violence if they fail to comply with the regime’s agenda.

Little is known for certain about the inner circle of decision makers in the context of war. Putin’s propaganda film, “Crimea: The Road Home” [*Krym: Put’ domoi*] which premiered in March 2015, tries to maintain that Putin made the decision to take Crimea with just four men: Nikolai Patrushev (secretary of the Security Council), Alexander Bortnikov (head of the FSB), Sergei Shoigu (Minister of Defense), and Sergei Ivanov (chief of staff). It seems impossible that an invasion of this magnitude and complexity could have been planned by just four men. Elaborate contingency plans had surely been drawn up months, if not years, in advance. Nonetheless, the fact that four of the five men had KGB training and leadership (only Shoigu did not) suggests that it is both the uniformity of the top circle and especially their involvement in secret services that is determinative (Howard and Pukhov, 2014; Zygar, 2016; Bukkvoll, 2018). Neither the military generals nor the Ministry of Foreign Affairs were consulted, if we are to believe Putin. In both 2014 and in 2022, Sergei Lavrov (head of the Ministry of Foreign Affairs) looked terribly surprised at what was happening. The Security Council of Russia, tasked with handling national security, has an entirely male leadership and only one woman *ex officio* who serves as a member (Valentina Matvienko, to whom we shall return below) (Sovet Bezopasnosti Rossiiskoi Federatsii, 2024).

The tiny number of women serving in top positions in government (as opposed to women parliamentarians, about which more below) have principally functioned as technocrats, working to make the polity run more effectively and/or in “social” positions relating to health and welfare. Most have served in executive rather than policy-making positions, and almost all of them have had Soviet-era education in economics, a rather narrow technical field usually associated more with accounting than policy-making.^4^ Elvira Nabiullina may be the one exception as the head of the Central Bank where she has overseen Russian monetary policy since 2014 (Prokopenko, 2022). Her role in stabilizing the economy and preventing panic in the Russian business community may itself play on a stereotype of women as managers and the backbone of the family (Nelson, 2022).

### The Russian National Guards, Putin’s personal guards, and Putin’s shadow paramilitary groups

Both in the taking of Crimea in 2014 (Howard and Pukhov, 2014; Wood, 2016b) and in the period between 2014 and 2022 (Bryjka, 2022; Potočňák and Mareš, 2022), *irregular* armed forces played a major role in destabilizing the internal situations and in preparing the way for the regular Russian military to intervene in 2022. It is thus important to see how they fit into the masculine structures of the regime.

The Russian Presidential Guard [*Rosgvardiia*] is the most recent branch of the Russian military forces, added in April 2016 to report to Putin himself without being integrated into the Russian armed forces. Highly conservative and hierarchical, Rosgvardiia initially drew the majority of its forces from the Ministry of Internal Affairs with leadership from veteran leaders of that ministry and the FSB (Bershidsky, 2016; Gresh, 2020). According to Bershidsky, it has its own intelligence branch, the authority to grant firearm licenses to individuals outside the military, and the right to fire without warning and make arrests without introducing themselves. While the Guards allegedly employ some 85,000 women (out of 350,000 total), only 20,000 are doing military service and they are primarily used to make traffic stops (TASS, 2018; Rabota v Rosgvardii, 2019). Many have had to sue to get in, and even then, not all have been successful (Piatyi Kanal, 2018; TASS, 2020). In the popular press, they tend to be celebrated for their participation in beauty contests (NEWS.ru, 2021). A recent newspaper article about three women guards referred to them as “girls” even though they had served for over 10 years and had children and husbands of their own (Kariakina, 2021).

In 2016 when he created Rosgvardiia, Putin chose to give the top directorship to Viktor Zolotov, his bodyguard from his years in the Leningrad mayor’s office (1991–1998) and the long-time head of his personal security service. A rather mysterious force, Zolotov has frequently been cited for his tight connections with Russian underworld boss, Roman Tsepov, as well, of course, as with Putin himself (Anin, 2018a; Zakharov and Badanin, 2020a,b). He is reported to have been at the meeting between Putin and Prigozhin [as well as Sergei Naryshkin, head of the Foreign Intelligence Service] after Prigozhin’s aborted uprising on June 23–24 (Osborn and Trevelyan, 2023).

Zolotov, his son and son-in-law and a number of Putin’s top personal bodyguards (all men) have been extensively rewarded with dacha land holdings near Putin’s Novo-Ogaryovo estate outside Moscow. At least six bodyguards have been promoted to positions of national authority (Anin, 2019).^5^ On May 25, 2022, the Kremlin promoted Aleksandr Kurenkov, another guard, to direct the Ministry of Emergency Situations (which had been under Shoigu from 1991 to 2012) (Radio Svoboda, 2022; Stewart, 2022; The Economic Times, 2022). As Russia expert Lilia Shevtsova once noted, this is “a praetorian regime run by people from the secret services” (cited in Rivera and Rivera, 2018, p. 223).

Meanwhile behind those official structures, the regime has encouraged a number of shadowy para-organizations with all-male or virtually all-male memberships and misogynist agendas. Recent news of Prigozhin’s uprising has made much of the Wagner group, but it is not the only one. Others include the Slavonic Corps (predecessor to Wagner), E.N.O.T., Patriot, and Shchit [Shield], the last two of which are offshoots of Wagner (“younger brothers,” according to Potočňák and Mareš, 2022, p. 194). All of these groups have actively recruited former combat veterans (many from the Afghan War) and members of all-male organizations such as the Russian Combat Brotherhood [*Boevoe bratstvo*], a veterans’ organization founded in 1997, but most influential from 2005 when it began to receive an influx of Kremlin money and patronage. Other volunteer groups based in male bonding have included Cossack societies in Crimea and southern Russia (which Russian authorities had been encouraging since the 1990s); the Union of Donbas Volunteers (created in 2015 by Putin’s then right-hand consultant, Vladislav Surkov); and the Volunteer Society for Cooperation with the Army, Aviation, and Navy (DOSAAF, a Soviet organization dating from 1927 and the only one that includes women as well as men) (Wood, 2016b). All of these groups have recruited through military commissariats dispersed across Russia that especially seek recruits with combat background from the Special Forces, the Airborne Forces, and Signal troops (Sukhankin, 2019; Chesnut 2020; Polezhaev, 2021). The Cossacks, in particular, have a highly masculine hierarchy (women may join the associations but may not attend the decision-making council) and serve as irregular militarized units that report directly to the Russian President; they can be mobilized by him overtly or covertly (Mineev, 2016; Darczewska, 2018).

In June 2023, Prigozhin publicly insisted that “as long as the special military operation is going on, men should be the ones to handle it” (Rasulova, 2023). The Wagner PMC, he noted, not only did not have any women in their forces; they would not take any as long as the war was ongoing. At the same time some women who have served in the Russian military forces have complained of the sexual harassment and violence they have experienced in those forces (Starikov, 2023).

One way to visualize the degree of male-centrism in these military and paramilitary forces is to look at photographs and footage of ceremonies in which Putin awards medals and honors. The highest award in the whole country is the Hero of the Russian Federation, created by President Boris Yeltsin in 1992 and awarded to Prigozhin in June 2022 (NGS, 2023). As of July 12, 2023, 1,293 individuals had received the award, 564 of them posthumously. Of those 1,293, only 19 [1.5%] have been women, and 10 of those 19 have been awarded posthumously, with the majority (13) awarded by Yeltsin. Only six have been awarded by Putin to women: two posthumously for WWII service, one posthumously for service in the Second Chechen War, one for a milkmaid (very Stalin-esque) who saved farm livestock from a fire; one for a Cosmonaut; and just one, posthumously, for service in the current Russo-Ukrainian War (in 2022) (Geroi Rossiiskoi Federatsii, 2024).

Looking at the official footage of the award ceremony on December 9, 2022 (the traditional date for Heroes of the Fatherland), two things immediately become obvious: (1) there are virtually no women present—even in the audience, and certainly none on stage to receive awards. This is a ceremony when men congratulate men, even if occasionally they give an award to a woman milkmaid. And (2) it appears that Prigozhin must have gotten the award in June 2022, rather than on December 9, the traditional date for that ceremony, which raises questions why he would have received the award off-cycle, as it were (Sputnik, 2022).

The group most openly hostile to women’s inclusion is undoubtedly the Night Wolves Motorcycle Club led by Alexander Zaldostanov, nicknamed “The Surgeon.” The Club has pursued a policy of excluding women since its founding in 1989. In an interview in August 1999, Zaldostanov explained that the Club had strict rules as to who could not join: “alcoholics, drug addicts, gays, and women.” He then elaborated: “A woman by virtue of her physiological particularities cannot be equal to a man—that’s just how it is. She has to know her place and not join in men’s games.” He would never give women positions of authority, he noted, because “you could not trust a woman’s logic and explain her actions” (Khirurg, 1999). In 2015, the Night Wolves and the Combat Brotherhood received large grants of state funds to support their so-called “Anti-Maidan” movement (Vedomosti, 2015). The Putin administration thus gives its largest funds to all-male organizations promoting militant sentiment without transparency and accountability to the population at large.

### Alpha male dominance in action

Putin’s alpha male dominance was particularly on view three days before the full-scale invasion of Ukraine when Putin called together his top leaders from the Security Council (at a distance of some 30 ft) to acclaim his decision to “recognize” the Donets and Lugansk “republics.” In a classic personal attack [*naezd*, in Russian], Putin established his dominance by speaking over his Foreign Intelligence director, Sergei Naryshkin, forcing him to answer “Yes or no?” until he gave the “right” answer that Putin sought. Throughout the show, Putin maintained a posture of casual dominance—hands on the arms of his chair as he slouched, showing his power over his subordinate (Lotareva and Golubeva, 2022; Vlamis and Haltiwanger, 2022).

Like fraternity brothers in American colleges, Russian men in politics and business have accepted this hazing under Putin because it has meant they are part of the club. Perhaps, the most famous example of an oligarch publicly accepting Putin’s domination came about when Putin arrived in the town of Pikalyovo in 2009 to resolve a labor dispute. As later scholars have noted, the solution to the conflict had already been found in Moscow (Fortescue, 2009). But Dmitry Medvedev was president, so Putin had to show that even though he was Prime Minister (a lesser position), he was still Boss no. 1. So, he personally came to town and roundly castigated all the participants (both management and protesters). The piece de resistance came when he called Oleg Deripaska, the owner of the local company and one of Russia’s richest men, to task, first for failing to sign the agreement and then for failing to return a pen that Putin had given him to sign it. By his tone, Putin made it clear that he was Boss, that he could attack any and all of the participants from the workers to the top management (Putin, 2010).^6^

Why would Deripaska accept such treatment? In part because he undoubtedly felt that he had no choice. The media made a special point of showing him with bowed head standing next to Putin. At the same time, though, it is important to remember that at the end of the day he received a $4.5 billion loan from the government. His closeness to power meant that he would continue to receive the choicest pieces of the economy, as long as he agreed to let Putin dominate. The media, in turn, chose to show his closeness to power and his abasement by Putin as a way of showing the latter’s power and control over his subordinates, his role as the just Tsar who could mete out rewards and punishments (Arutunyan, 2014, 67–83).

In all these situations, Putin’s relations with those around him demonstrate a heavy dose of implied violence, boorishness [*khamstvo*], and the humiliation, both of foreign journalists and of his own top Cabinet officials whom he treats as children whom he must instruct. This combination of “instruction” and violence has proved particularly corrosive to Russian democratic institutions, demonstrating personalist, male-dominated solutions to systemic problems without broad discussion and consensus. However much Putin might speak of the “rule of law” and say that it was highly irregular for him to come and personally resolve the situation in Pikalyovo, it was clear that this was a made-for-TV drama to keep Putin in the spotlight even when he was not sitting in the highest office in the land. While viewers might be glad of a “good tsar” on the throne, his “manual control” has meant that normally challenging situations of governance are solved not by the bodies of government nor through the application of law. The result is highly predictable, especially when everyone knows what Putin thinks about “traitors” and they know what happens to Putin’s “enemies” when they are poisoned or murdered in contract killings (Dewey, 2023).

## The self-subordination of the Russian Parliament

In January 2013, Russia’s prominent TV presenter Vladimir Pozner “accidentally” referred to the State Duma, known in Russian as the GosDuma, as “Gosdura,” or state fool (Nadezhdin, 2013; Pozner, 2013). The term quickly took off in Internet memes. It was even voted “Word of the Year” (AdIndex, 2013). But “dura” is not just a fool; it is a woman who is empty-headed and silly.

Political scientist Julian Waller has argued that the deep and growing illiberalism of the Duma, i.e., its commitment to arch-conservative values, has its basis in what he calls the “entrepreneurial behavior by lower-tier elite signaling loyalty and usefulness to the regime center” (Waller, 2021, p. 1). By doing the regime’s bidding, they show off their commitment to the centralized and authoritarian political process as well as to Putin personally. In return they are allowed to keep their seats in the Duma, which give them apartments in Moscow, diplomatic immunity, and sizable rewards in land, money, and state contracts for their businesses.

Efforts to subdue the Duma seem to have intensified when Putin returned to power after 4 years of the Medvedev presidency (2008–2012). In part this was a response to the protests against Putin. From September 24, 2011 when Medvedev announced that Putin would again be the heir presumptive for the next term, protesters savaged Putin’s masculinity with images of condoms (after Putin had claimed that he thought the protesters’ white ribbons were part of an AIDS demonstration), memes of Putin “on top” in relationship with Medvedev, and songs and videos like “Our Nuthouse Votes for Putin” (Novitskaya, 2017; Ekşi and Wood, 2019).

Immediately after his return to power in May 2012, the Duma began spitting out anti-western laws on adoption, restrictions on keeping money abroad, bans on so-called “homosexual propaganda,” and the like.^7^ Russian journalist Andrey Pertsev (2022) has provided an excellent analysis of how the law banning foreigners from adopting Russian children was used to whip the Duma into shape. As he shows, the bill originated with the Russian Presidential Administration and the Security Council, but was then sent to State Duma Speaker Vyacheslav Volodin who passed it on to Ekaterina Lakhova. Lakhova, a co-founder of the Women of Russia Party in 1993 and a Duma representative since then, was to serve as the face of the bill. All those who disagreed with the bill were told that they would have troubles with their businesses and/or would be pushed out of office if they resisted. At his annual *Direct Line with Putin* at that time, the Russian President blew up at one reporter who criticized the law banning US adoptions, saying “You think it is normal if [the U.S.] humiliates us? Are you a sadomasochist or what?” (von Twickel, 2012). In fact, it was Putin who was humiliating his interlocutor, impugning his masculinity and showing his own dominance.

## The baba commissars

A distinct group of dominant women stand out in the Duma as movers and shakers, including Valentina Matvienko, Valentina Tereshkova (the former cosmonaut), Elena Mizulina, Irina Yarovaya, and Ekaterina Lakhova (among others). Each has promoted legislation that supports the Russian President, often appearing even more extreme than him in their support for anti-Western and conservative positions (Stolyarova, 2008). I have chosen to call them the “baba commissars.” In Russia historically the somewhat derogatory term “baba” meant a strong peasant woman (Viola, 1986; Wood, 1997). The commissar, meanwhile, was the one who provided the political education and control in the new revolutionary state after 1917. In the 1967 film *Commissar* [Komissar, in Russian], the main character Klavdiia Vavilova, shows her toughness as a female commissar with the Red Army cavalry in an opening scene when she violently chews out a man who has left his unit to go visit his family. Her stream of invective qualifies her as “man.” Her victim falls to his knees, and the men in her unit nod their recognition of her power. Once she has a child, however, her own men turn on her, calling her “grazhdanochka” (a little miss, not a comrade) and “Vavilovka [little Vavilova].”

Numerically women currently have fairly low representation in the lower house of Parliament, the Duma, and in the upper, the Federation Council. As of 2023, women occupied 74 of 450 seats in the Duma (16.4%) and 36 out of 169 seats in the Federation Council (21.3%). In this they ranked 138 out 185 countries in the world.^8^ The Federation Council (the upper house) does have a well-known woman chair, Valentina Matvienko. In the Duma, the Parliament elected from 2016 to 2021 had three women deputy speakers out of 11 (Irina Yarovaya, Olga Timofeeva, and Olga Yepifanova). The Duma elected in 2021, by contrast, had only 2 deputy speakers out of 11 (Anna Kuznetsova and Irina Yarovaya) (List of Deputy Chairmen of the State Duma, 2022).

Analysis of the leading women politicians suggests that they fall into two distinct generations: (1) the oldest women (Matvienko [b. 1949]; Tereshkova [b. 1937]; Lakhova [b. 1948]), born and raised entirely in the Soviet Union, together with a slightly younger Soviet cohort (Svetlana Orlova [b. 1954], Mizulina [b. 1954], Tatiana Golikova [b. 1966]; Yarovaya [b. 1966]; Olga Golodets [b. 1962]); and (2) the younger generation (Anna Kuznetsova [b. 1982]; Maria Lvova-Belova [b. 1984]). Almost all of the most prominent women parliamentarians have played disproportionate roles in initiating and championing legislation that is controlling toward society—both policies on “the family” and legislation on foreign agents and censorship.

It has usually been assumed that these women are merely doing the regime’s bidding in return for a seat at the table. That is definitely part of the story. Johnson (2018, pp. 72-73, 79-83) has added more nuance by suggesting four categories of women politicians: “workhorses” who strive to perform without much fanfare; “political cleaners” who are recruited for their appearance of being less corrupt; “loyalists” who strive to protect the regime; and “showgirls,” i.e., beautiful women who attract voters by their celebrity and good looks. The challenge with most work on women in Russian politics is that it has been fixated on the “glass ceiling.” Johnson (2016, 2018) has argued that while women may have been “fast tracked” into politics at a few key moments in time, once there, they end up “boxed in,” unable to advance further. But this research has not asked *what is the work that these women are doing for the regime? Why does the regime need these strong women who pass legislation that is not always in women’s own interests?* If, like John F. Kennedy, we ask not what their country is doing for them, but rather what they are doing for their country, we see that they play a critical role in enhancing the narrative of Russia as a besieged fortress, a nation under threat because of the social policies and practices of other nations and peoples.

In terms of what they personally obtain by serving in the Duma, those advantages are not different from those of their male counterparts: political, social, and economic perquisites, and the appearance of power. But what they give the regime is a particular kind of *support*, and also a *cover*, that is deeply gendered. In trying to prove their own loyalty and closeness to power, they provide multiple kinds of legitimacy to a system that is otherwise based on force, violence, and insider connections. We can see how this works in multiple ways.

First, in their pronouncements they frequently claim that they represent grassroots supporters. Implied in this is an assumption that because women are not the actual figures of power [*vlastiteli, praviteli, rukovoditeli*], their support proves that the masses support the regime. Such grass roots support continues a Soviet tradition of maintaining that this is a people’s regime supported by the broad masses. Their “popular” support also resembles that of the Gorbachev-era chemistry teacher, Nina Andreeva, whose essay “I Cannot Forsake My Principles” frontally attacked *perestroika* and *glasnost* when it was published in the right-wing newspaper *Sovetskaia Rossiia* in March 1988. In February 2023, Matvienko claimed a similar popular mandate, saying “The people of Russia are united” in resisting Western threats (Matvienko, 2023; RIA Novosti, 2023).

Secondly, the baba commissars are all women who have been chosen to represent a deeply conservative picture of society, especially since Vladimir Putin’s own turn toward conservatism in 2012. These are not liberal women, though a few of them did begin their political careers in liberal politics, especially the Yabloko party (RBK, 2019).^9^ These are women who, like the recent Minister of Education Ol’ga Vasilieva, have openly expressed admiration for Brezhnev and even for Stalin (International Business Times UK, 2016; Pravmir, 2016). They have pioneered the most illiberal legislation on adoption (as mentioned above), abortion, domestic violence, homosexuality, and control over the Internet.

Thirdly, this conservative view, in turn, puts the church and state in line with each other where each can benefit from their ideological connection. This creates a space for women as guardians of private *and public* morality. And it proves the need for such guardians because it mobilizes a *threat narrative* of gays, equated with pedophiles, emerging out of every closet.

Fourth, the performances of the baba commissars play into a long history of late Tsarist and Soviet-era tropes of strong women who support the regime. Examples of these strong women range from the Women’s Battalion of Death created by the Provisional Government in 1917 for shaming men into joining the armed forces to women’s roles as the “sharp eyes and tender hearts” of the Revolution fighting corruption in the dining halls (Wood, 1997; Stockdale, 2004; Stoff, 2006). In WWII and after, women were celebrated as sharpshooters and snipers, “night witches” (pilots), and femmes fatales in the counter-intelligence SMERSH battalions which shot deserters at the front lines (Krylova, 2004, 2011; Rossiiskaia gazeta, 2006; Harris, 2008). In all these contexts and many others, women played a key role in enforcing Soviet norms and values. While women are not the only “moral crusaders” in the Duma today, they do play an outsized role on committees that claim to protect children and society as a whole (Sharafutdinova, 2014).

In all these ways, these women parliamentarians consistently motivate their pronouncements on the grounds that they are *protecting society*, demanding a strong state to defend the nation against invisible but omnipresent threats both from outside and from inside. In the period after Putin’s return to the presidency in 2012 when the Duma’s legislation had gone so wild it was known as “the mad printer,” these women had a special role to play in enforcing subordination to the top powers. Together, the Matvienkos, Mizulinas and others have acted as the “mothers” of the protectionist political order. They are not the decision-makers for security matters of war and peace. Rather they are the enforcers who amplify the directions taken by the male political elites.

It might be asked, of course, if any of these women have real power. Political observers, both academics and journalists, sometimes hypothesize that Matvienko might be in Putin’s inner circle, one of the few people that Putin consults in moments like the invasions of Crimea and Donbas (in 2014) and the full-scale invasion of the nation of Ukraine on February 24, 2022. Yet she is on record as saying—on February 16, 2022 (!)—that even the thought of war between Russia and Ukraine would be “heretical” given the long relations between the two peoples (Federation Council, 2022; Lenta, 2022; TASS, 2022c). It seems unlikely that she was part of the “inner circle” making the decision in this case. But she was ready to serve as an enforcer. Just 5 days later, on February 21, 2022, Matvienko began to browbeat other deputies into supporting Russia’s “annexation” of Donetsk and Luhansk. It was Russia’s “moral duty” to “recognize” the annexation of the two republics, she insisted. Claiming the emotional high road, Matvienko continued with tears in her eyes: “In the 30 years of its separate existence [Ukraine’s], how has Russia ever harmed Ukraine, what harm has Russia brought?” (Lotareva and Golubeva, 2022).

This reduction of the Duma to puppet status and the prominent role of women as enforcers means that Putin and his small inner circle did not face political resistance from the legislative branch when they undertook the invasion of Ukraine. New laws annexing the occupied territories were passed almost instantaneously. The fiction of popular support could be maintained at the highest level because women deputies appearing to represent “the people” gave their loud acquiescence. In the atmosphere of anxiety whipped up through laws about pedophiles and invasive social movements from Gay Europe [*Gayropa* in Russian], a fearful mentality about supposed Nazis and Western invasion could easily be transmitted to the general population. In June 2023, two women leaders of the Duma, Anna Kuznetsova and Galina Karelova, even co-founded a “Parliamentary Commission for the Investigation of Criminal Actions against Minors by the Kyiv Regime”! (Website of the State Duma, 2023a,b).

## Concluding thoughts

The gender picture, like everything else about the ruling structures of Putin’s Russia, is anything but simple. The tough men who form the inner circle of Putin’s regime rely on violence, extortion and blackmail, backroom deals, and mafiosi-style protection rackets to keep themselves in power. Putin’s bodyguards and his “private” military contractors receive lavish rewards as long as they stay in line. As the stories of Prigozhin and others who criticize the military for not being macho enough reveal, some criticism can be tolerated and even encouraged by the regime, as long as it involves males shaming other males into behaving. Yet there are always limits, and Prigozhin and his comrades ended up paying a stiff price for their views challenging the alpha male gender order.

For public consumption, the president also has a small army of women who pass legislation focused on controlling the Russian polity against outside threats—homosexuality, foreign values, and practices. In the public eye, they are viewed as tough and uncompromising. While the men make arguments about NATO, militarization and Nazis, the women claim that they have to protect Ukrainian children in the Donbas and help them receive care in Russia (Karelova, 2023).

At the end of the day, it is doubtless impossible to say which came first, the male-centered cadres at the top of the hierarchy or their mafia-style administration of instruction and violence. Almost certainly, the two have been mutually reinforcing since Vladimir Putin first came into the highest offices in August 1999. At that time, he particularly sought to dominate since he was coming in as an outsider who needed to build up his own cadres. The late 1990s were also a time of extensive violence among business elites themselves (Soldatov and Borogan, 2010; Volkov, 2016). It is difficult to say whether such a combination of male domination and selective use of violence outside the normal parameters of state control automatically heralds a potential for war and invasion. But at the end of the day, the Putin-era systems of governance have become deeply intertwined with the use of military force without regard for international conventions on humanitarian relations between nations and peoples and with systematic disregard for ordinary human morality and ethics.

## Data availability statement

The original contributions presented in the study are included in the article; further inquiries can be directed to the corresponding author.

## Author contributions

EW: Writing – original draft.

## Footnotes

^1^Most of the recruiting centers are in fight clubs, martial arts schools, and boxing clubs, typically entirely male spaces.

^2^For arguments that Putin has not, in fact, become more conservative in his gender statements but has, rather, maintained a steady Soviet understanding of gender up until 2020, see Johnson et al. (2021).

^3^Kolesnik (2022) calculated that 83% were classified as men and only 17% as women.

^4^Those with Soviet-era economic or accounting degrees include Elvira Nabiullina, chair of the Central Bank of Russia since 2013; Tatyana Golikova, Deputy Prime Minister of Russia for Social Policy, Labor, Health and Pension Provision since 2018; and Olga Golodets, Deputy Prime Minister from 2012 to 2020. Even Tatyana Shevtsova, Deputy Minister of Defense, received her undergraduate and graduate degrees in economics, and she worked exclusively in the Federal Tax Service of the Russian Federation before becoming Deputy Minister of Defense in 2010. Ella Pamfilova, chair of the Central Election Commission, has a decree in electronics. Olga Vasilieva, Minister of Science and Education (2016–2020), came to government as a church choir director and proceeded to extol both Stalin and Stalinist values. Of the 83 heads of Russia’s so-called federal subjects, i.e., the country’s republics, territories, oblasts, and the capital cities of Moscow and St. Petersburg, only one is a woman. Natalya Komarova, the governor of Khanty-Mansiisk and a member of the ruling United Russia Party, is also the oldest of all the governors (born in 1955) (Deistvuiushchie glavy sub”ektov Rossiiskoi Federatsii,”, 2023).

^5^Other bodyguards who have received important promotions: Alexey Dyumin, 49, governor of Tula region; Dmitry Mironov, 53, now an assistant to Putin after a stint as governor of Yaroslavl region; Sergei Morozov, 49, governor of Astrakhan region before returning to the secret services as a Major-General (Anin, 2018b).

^6^The official Russian press also singled out Putin’s treatment of Deripaska as the most important part of the whole encounter (RT, 2009; Tsyganov, 2009).

^7^For a humorous overview of all the “anti-” legislation see Bohm (2014). For more on this, see Waller (2023), Sleptcov (2018), and Sharafutdinova (2014).

^8^“Monthly ranking of women in national parliaments,” https://data.ipu.org/women-ranking?month=7&year=2023. Statista has 16.7 and 22.3%, respectively, in data taken from Russian Federal State Statistics Service (March 1, 2023). Share of seats held by women in national parliaments in Russia from January 1, 2014 to January 1, 2023, by institution. Retrieved July 24, 2023, from https://www-statista-com.libproxy.mit.edu/statistics/1123939/share-of-women-in-parliaments-in-russia/. In both cases, this represents data from the September 2021 elections to the Duma. The Federation Council members are appointed, not elected. Initially, they were supposed to be elected so as to represent the regions of Russia. This, however, has become less and less true over time (Ross and Turovsky, 2013).

^9^Lakhova began in the Committee on Women, the Family and Children as a member of the Communist Party in 1990, then helped to found “Women of Russia” in 1993. Mizulina was first elected to the Duma from the Yabloko (liberal) party in 1995. One more mixed figure has been Oksana Pushkina who mostly played a conservative role in the Duma (2016-2021) as a “TV star” and Vice-Chair of the Committee on the Family, Women and Children. But she also openly told the press about violence against her and others over a new law on family violence, and she backed women’s complaints against Leonid Slutsky, accused of sexual harassment (Pushkina, 2018; RBK, 2019).
